# Enhancing the Reliability of Cartilage Repair Evaluation: A Simplified Volume‐Based Approach

**DOI:** 10.1002/jor.26096

**Published:** 2025-05-11

**Authors:** Jani Puhakka, Teemu Paatela, Eve Salonius, Virpi Muhonen, Anna Meller, Anna Vasara, Hannu Kautiainen, Jussi Kosola, Ilkka Kiviranta

**Affiliations:** ^1^ Department of Orthopaedics and Traumatology University of Helsinki Helsinki Finland; ^2^ Spine Center Schulthess Clinic Zurich Switzerland; ^3^ Laboratory Animal Center, Helsinki Institute of Life Science (HiLIFE) University of Helsinki Helsinki Finland; ^4^ Primary Health Care Unit Kuopio University Hospital Kuopio Finland

**Keywords:** animal model, arthroscopy, cartilage repair, ICRS, preclinical, reliability, scoring system

## Abstract

**ABSTRACT:**

The purpose of this study was to explore methods for enhancing the reliability of arthroscopic cartilage repair evaluation. We compared a new volume‐based scoring technique that assessed the extent of high‐quality cartilage repair tissue in four quadrants of the repair tissue with the International Cartilage Repair Society (ICRS) score. Using a porcine cartilage repair model, we evaluated the inter‐ and intrarater reliability of the new volume‐based technique. Nine defects were treated with a recombinant human Type III collagen/polylactide scaffold, whereas nine were left to heal spontaneously. The reliability of the volume‐based score was analyzed using intraclass correlation coefficients (ICCs), and external validation was performed by comparing the score with histological ICRS II results. The volume‐based score demonstrated moderate to good interrater reliability (ICC 0.67–0.78) and intrarater reliability (ICC 0.58–0.84), outperforming the ICRS score in consistency. Moderate positive correlations were observed between the volume‐based score and histological ICRS II subscores (*r*_s_ = 0.62–0.64, *p* < 0.001). These findings suggest that the volume‐based approach may improve the reliability of arthroscopic cartilage repair assessment without significantly compromising the alignment with histological evaluations. Although the simplified system offers advantages in reproducibility, further refinement is necessary to balance reliability with the need for comprehensive tissue evaluation.

**Level of Evidence:**

Diagnostics, Level III (based on comparative studies in an animal model with blinding between observers).

**Statement of Clinical Significance:**

This study provides insights into improving the reliability of arthroscopic cartilage repair evaluations, potentially leading to more consistent and accurate assessments in both clinical and research settings.

## 1 Introduction

Accurate evaluation of cartilage repair is essential for assessing emerging treatment techniques, particularly when clinical outcomes remain to be established. Several arthroscopic classification systems have been developed for this purpose, among which the International Cartilage Repair Society (ICRS) scoring system is widely used. The ICRS system—validated in previous studies—evaluates repair tissue based on three key aspects: tissue fill, integration with the surrounding cartilage, and macroscopic appearance [1, 2]. Specifically, “repair tissue fill” measures the extent to which a defect is filled with new tissue; “integration” assesses how seamlessly the repair merges with adjacent cartilage; and “macroscopic appearance” compares the repair tissue to normal hyaline cartilage.

The arthroscopic ICRS scoring system has shown a moderate correlation with histological findings when compared with the more detailed ICRS II scoring system, which comprehensively evaluates tissue characteristics through multiple parameters (e.g., tissue structure, matrix staining, and cell distribution) [3]. However, several studies have highlighted significant limitations in the reliability of these systems, particularly when used in larger, heterogeneous lesions [4, 5]. In these cases, repair tissue often only partially fills the defect, resulting in uneven and inconsistent tissue quality that may undermine the accuracy of the scoring systems [6].

In particular, Brittberg and Winalski emphasized the importance of assessing the volume of the defect filled with repair tissue, yet current methodologies offer limited guidance on how to evaluate lesions that are only partially filled [1]. The ICRS system, for example, is more appropriate for evaluating homogeneous repair tissue, and assessments of partially filled and heterogeneous repair tissue introduce variability. This variability affects both inter‐ and intrarater reliability, as evaluators may focus on different regions of the lesion, leading to inconsistent results.

To address the limitations of current cartilage repair evaluation methods, focusing on the volume and distribution of repair tissue across the entire defect site may be beneficial. Existing detailed scoring systems often face challenges with reproducibility, particularly when assessing the heterogeneity of repair tissue, as they rely on qualitative evaluations of multiple attributes. Simplifying the evaluation process by emphasizing volumetric assessment—determining how much of the defect is filled with high‐quality repair tissue—could potentially reduce variability and improve consistency. Evaluating the entire defect as a whole, rather than relying on detailed point‐based scoring, may offer a way to account for the heterogeneity inherent in repair tissue.

The purpose of this study was to evaluate whether a new arthroscopic scoring system for cartilage repair, which assesses cartilage as a volume and simplifies the evaluation of repair tissue quality, could enhance reliability in terms of inter‐ and intrarater consistency. We compared the reliability of a simplified volume‐based scoring system with the established ICRS score to assess inter‐ and intrarater reliability. Additionally, external validation was conducted by correlating the volume‐based scores with histological ICRS scores to determine their accuracy. Our findings aim to contribute to ongoing efforts to address the challenges of evaluating cartilage repair tissue by emphasizing the importance of consistent and reproducible scoring methods.

## 2 Materials and Methods

### 2.1 Experimental Animals and Ethical Considerations

Eighteen 4‐month‐old female domestic pigs (*Sus scrofa domestica*, *n* = 18) were obtained from a local farm for this study, with the use of a single sex chosen to minimize variability due to hormonal and anatomical differences. We determined our sample size by assuming an expected ICC of 0.7. According to Bonett, 15–20 subjects are needed to achieve a 95% confidence interval (CI) with a half‐width of about 0.15–0.20 [7]. All animals were acclimatized to the experimental facility for 14 days before the surgical procedures. During this period, they were housed in group pens with free movement and bedding, under veterinary supervision. The study was conducted according to the ethical guidelines of the Finnish Act on the Protection of Animals Used for Scientific or Educational Purposes (497/2013) and was authorized by the National Animal Experiment Board (ESAVI/6113/04.10.07/2015).

### 2.2 Surgical Procedure

For preoperative analgesia, each animal received 0.05 mg/kg buprenorphine, 3 mg/kg carprofen, and 3.0 mg/kg cefuroxime. Anesthesia was induced with 0.2 mg/kg medetomidine and 10 mg/kg ketamine, followed by 3 mg/kg propofol, and maintained with 1.5%–2.5% isoflurane. After intubation, the animals were placed in the supine position on the operating table. A medial parapatellar arthrotomy was performed on the right hind leg to dislocate the patella laterally, exposing the weight‐bearing surface of the medial femoral condyle.

**Table 1:** Macroscopic evaluation of cartilage repair with the International Cartilage Repair Society (ICRS) scoring system [1].

Cartilage repair assessment, ICRS	Points
Degree of defect repair	
Level with surrounding cartilage	4
75% repair of defect depth	3
50% repair of defect depth	2
25% repair of defect depth	1
0% repair of defect depth	0
Integration with border zone	
Complete integration with surrounding cartilage	4
Demarcating border < 1 mm	3
3/4 of graft integrated, 1/4 with a notable border > 1 mm width	2
1/2 of graft integrated with surrounding cartilage, 1/2 with a notable border > 1 mm	1
From no contact to 1/4 of graft integrated with surrounding cartilage	0
Macroscopic appearance	
Intact smooth surface	4
Fibrillated surface	3
Small, scattered fissures or cracks	2
Several small or few large fissures	1
Total degeneration of grafted area	0
Overall repair assessment	
Grade I: Normal	12
Grade II: Nearly normal	11–8
Grade III: Abnormal	7–4
Grade IV: Severely abnormal	3–1

To create standardized, full‐thickness cartilage defects, a custom hollow punch (11 × 17 mm) was used, based on pilot experiments confirming the safety and consistency of this approach. The oval‐shaped defect was created without removing the underlying subchondral bone. After the procedure, the animals were allowed unrestricted movement and normal weight‐bearing. Postoperative care included 3 days of analgesia (buprenorphine and carprofen) and antibiotic prophylaxis (cephalexin).

Five weeks later—allowing sufficient time for early repair tissue formation—the animals underwent a second operation to debride the defect area down to the subchondral bone.

Nine pigs received a recombinant human Type III collagen/polylactide scaffold [8, 9], which was fabricated by combining a biodegradable polylactide matrix with recombinant human Type III collagen to support chondrogenesis. The scaffold was cut to fit the defect and secured with absorbable sutures (Monocryl 6–0, Ethicon). The remaining nine pigs served as controls and were left to heal spontaneously after debridement. Following the second operation, the animals were allowed unrestricted movement to simulate natural weight‐bearing. Healing was monitored via regular clinical examinations and behavioral observations. Postoperative care included a 3‐day treatment with cephalexin for antibiotic prophylaxis and the same analgesic regimen used after the first procedure. All surgical procedures were performed by two experienced orthopedic knee surgeons (A.V. and T.P.), both with extensive experience in large animal models and cartilage repair research.

### 2.3 Simulated Arthroscopic and Video Evaluations of Cartilage Repair

**Figure 1:**
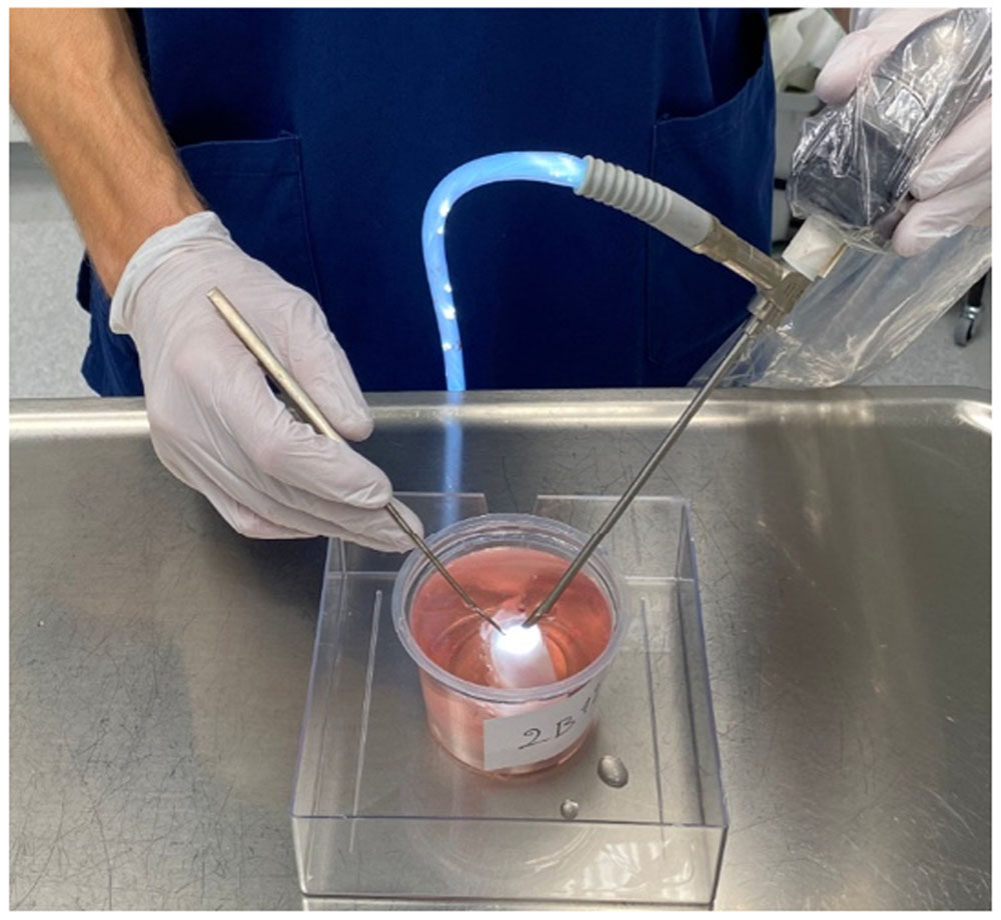
Simulated video‐recorded arthroscopy with a camera and a probe.

Four months after the initial surgery, the animals were euthanized using intravenous anesthetic, and the medial femoral condyles were harvested. The excised specimens were placed in a phosphate‐buffered saline (PBS) solution containing 5 mM ethylenediaminetetraacetic acid (EDTA) disodium salt (VWR International) and 5 mM benzamidine hydrochloride (Sigma‐Aldrich), which inhibited metalloprotease activity. The specimens were secured in containers and immersed in PBS solution to simulate in vivo arthroscopic conditions (Figure 1).

Arthroscopic evaluations were performed using a standard arthroscopy tower (Karl Storz Endoscopy) equipped with a 4.0 mm, 30° angled optic, and a standard arthroscopic probe. The procedures were video‐recorded for subsequent evaluations. Two independent evaluators assessed the repair tissue: an orthopedic resident with 1 year of arthroscopy experience (“Resident,” E.S.) and an orthopedic fellow with 6 years of experience (“Fellow,” J.P.). Both evaluators had prior experience using the arthroscopic ICRS scoring system in clinical and laboratory settings, including cartilage repair studies involving scaffold models, and were familiar with histological ICRS II scoring methods. Importantly, earlier findings suggest that the reliability of the ICRS scoring system is only minimally influenced by evaluator experience [5]. Evaluators were blinded to both the treatment group (scaffold vs. control) and each other's results.

### 2.4 Evaluation of Cartilage Repair

**Figure 2:**
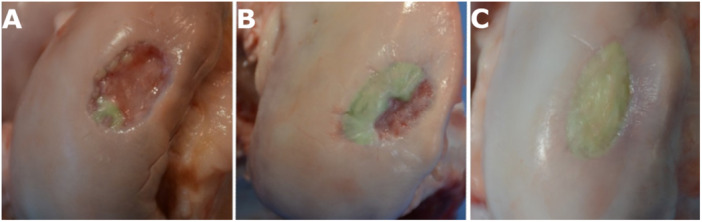
Example of volume‐based cartilage repair scoring. (A) Less than one‐quarter of the defect filled with high‐quality cartilage repair tissue, yielding a score of 0 points. (B) Approximately two‐quarters (> 50%) of the defect filled with high‐quality cartilage repair tissue, yielding a score of 2 points. (C) All quadrants of the defect filled with high‐quality cartilage repair tissue, yielding the maximum score of 4 points. *Note:* The areas considered to have high‐quality cartilage repair are highlighted with a green color filter to illustrate the quadrants being assessed. The green filter is for illustrative purposes and does not represent the actual color of the repair tissue during arthroscopy.

The evaluators scored each specimen using the arthroscopic (macroscopic) ICRS system (Table 1), a widely used and validated method for cartilage repair assessment, and the tested volume‐based method, allowing for a direct comparison of their reliability and applicability. The latter involved assessing how many quarters of the repaired area contained high‐quality cartilage‐like tissue, as determined by the evaluator's clinical judgment based on complete defect fill, macroscopic appearance, and tactile assessment, yielding a total score from 0 to 4 (Table 2, Figure 2). All evaluators reassessed their recorded videos twice within 9 months of the initial evaluation, in a blinded and randomized manner, using a web‐based platform (SurveyMonkey, San Mateo, CA, USA). The first reassessment occurred 6 months after the initial arthroscopy, and the second occurred 3 months later. The evaluators were blinded to their prior assessments to minimize bias.

**Table 2:** Evaluation of cartilage repair using the volume‐based scoring system. This table shows the evaluation criteria for cartilage repair based on the volume‐based scoring system. The score is determined by the proportion of the defect area filled with high‐quality cartilage repair tissue, assessed in four quadrants of the defect.

Proportion of defect area repaired with good‐quality cartilage	Points
100% (all four quadrants repaired)	4
75% of the defect area (three quadrants repaired)	3
50% of the defect area (two quadrants repaired)	2
25% of the defect area (one quadrant repaired)	1
0% of the defect area (no quadrants repaired)	0

### 2.5 Histology

Histological validation of the arthroscopic assessments was conducted using previously published ICRS II scores from the same cartilage repair samples, which had been analyzed in earlier studies on the effectiveness of cartilage repair with and without scaffold‐based techniques, enabling a direct correlation between arthroscopic scoring and histological outcomes [3, 6]. Each medial femoral condyle specimen was bisected through the center of the defect with a diamond saw, and the resulting halves were fixed in 10% buffered formalin for 4 weeks. Samples were then decalcified in 10% EDTA solution, embedded in paraffin, and sectioned into 5‐μm slices. Safranin‐O staining was used to assess cartilage repair quality. Histological evaluation was performed using the ICRS II [10] scoring system by the same two surgeons (J.P. and E.S.) in a fully blinded manner, with samples reviewed in random order to minimize observer bias. Both surgeons had extensive experience in histopathological evaluation, and assessments were conducted independently, blinded to both the arthroscopic results and treatment groups.

### 2.6 Statistical Analysis

Three reliability metrics were used to evaluate the interrater reliability of the volume‐based score:

Internal consistency was measured using Cronbach's *α* coefficient, where values between 0.7 and 0.8 were considered satisfactory for research purposes, and values above 0.9 were ideal for clinical application [11].

Intraclass correlation coefficients (ICCs) were calculated using a two‐way random‐effects model for absolute agreement, based on single rater measurements. ICC values were interpreted as follows: below 0.5 indicated poor reliability, 0.5–0.75 moderate reliability, 0.75–0.9 good reliability, and above 0.9 excellent reliability [12].

To enable comparison between the ICRS score and the volume‐based score, we extracted the ICRS scores from a previous reliability study using the same repair tissue samples [5]. Normalization of the ICRS and volume‐based scores, as well as the histological ICRS II scores, was performed using the rank‐based normalization method of van der Waerden [13]. Spearman correlation coefficients were calculated between the normalized ICRS scores and the volume‐based scores, as well as between the histological ICRS II scores and both arthroscopic scores. Bonferroni‐adjusted *p*‐values were used for significance testing, and 95% CIs were reported. All statistical analyses were conducted using R Studio version 2023.03.0 + 386 (R Studio, Boston, MA, USA).

## 3 Results

The porcine cartilage repair model yielded heterogeneous repair tissue, ranging from poor to good quality across the specimens. The mean total score for the volume‐based evaluation was 2.2 (standard deviation [SD] 1.1) during the initial arthroscopy, 2.3 (SD 1.2) in the first reevaluation, and 2.3 (SD 1.2) in the second reevaluation. The scaffold‐repaired cartilage group produced slightly higher scores compared to the spontaneous repair control group, but these differences were statistically insignificant.

The internal consistency of the volume‐based scoring system, as measured by Cronbach's *α*, was 0.90 in the initial arthroscopy, indicating high reliability across the score items.

The interrater reliability of the score, expressed as the ICC, ranged from 0.67 to 0.78, showing moderate to good reliability during both the initial arthroscopy and the two subsequent reevaluations (Table 3). Similarly, the intrarater reliability demonstrated moderate to good agreement, with ICC values ranging from 0.58 to 0.84 across all assessments (Table 4).

**Table 3:** Interrater reliability of cartilage repair evaluation using the volume‐based score and the arthroscopic ICRS total score. This table presents the interrater reliability of evaluating cartilage repair using the volume‐based score compared with the previously published arthroscopic ICRS total score. Intraclass correlation coefficient (ICC) estimates and their 95% confidence intervals (CIs) are calculated using an absolute agreement, two‐way random‐effects model.

	Arthroscopy (95% CI)	Reevaluation (video) 1	Reevaluation (video) 2
Volume‐based score	0.77 (0.57–0.90)	0.78 (0.59–0.90)	0.67 (0.44–0.85)
ICRS total score [5]	0.60 (0.34–0.81)	0.57 (0.30–0.79)	0.46 (0.17–0.72)

**Table 4:** Intrarater reliability of cartilage repair evaluation using the volume‐based score and the arthroscopic ICRS total score. This table presents the intrarater reliability of the volume‐based score compared to the previously published arthroscopic ICRS total score. Intraclass correlation coefficient (ICC) estimates and their 95% confidence intervals (CIs) are shown for both scoring methods.

	Resident	Fellow	Consultant
Volume‐based score	0.84 (0.68–0.93)	0.58 (0.31–0.80)	0.83 (0.68–0.93)
ICRS total score [5]	0.54 (0.26–0.78)	0.52 (0.25–0.76)	0.59 (0.33–0.80)

**Figure 3:**
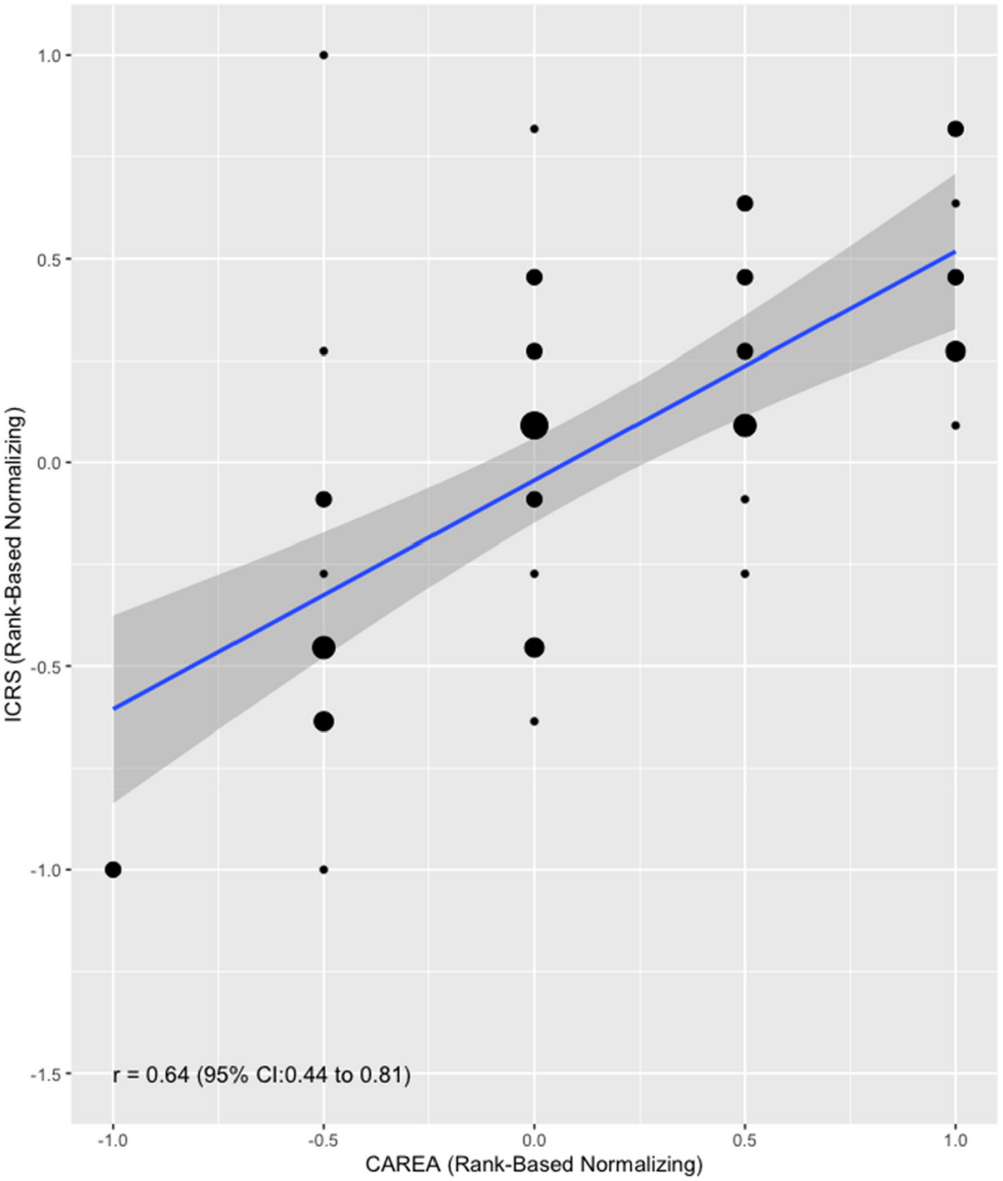
The plot shows the correlation between the arthroscopic ICRS score and the volume‐based cartilage repair score (CAREA), with a regression line and 95% confidence interval (gray area) included. The correlation demonstrates the relationship between the two scoring methods in assessing the quality of cartilage repair.

**Figure 4:**
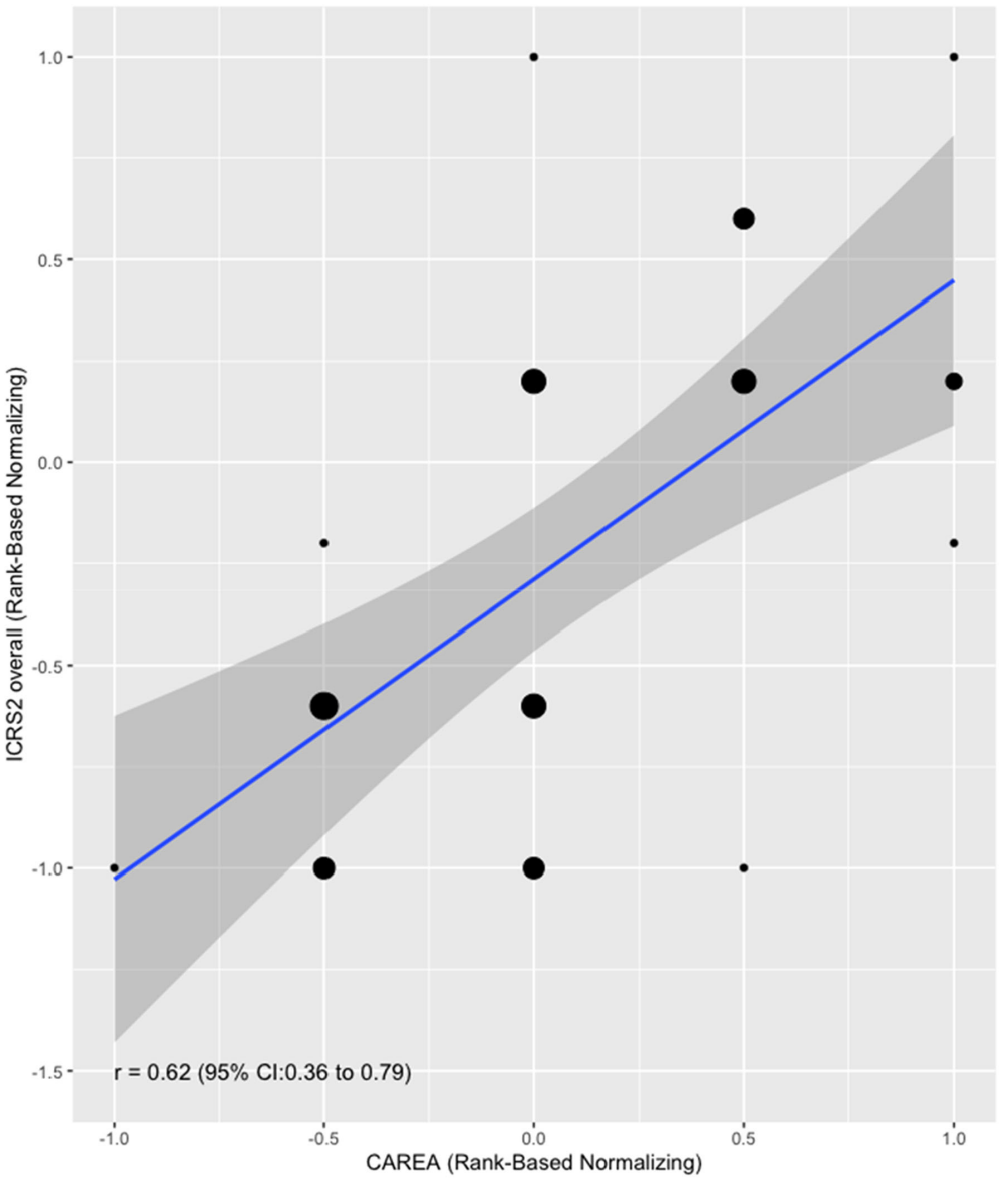
Correlation plot between the volume‐based cartilage repair score (CAREA) and the histological ICRS II scores, including both the overall assessment subscore and the average of all ICRS II subscores. A regression line and 95% confidence interval (gray area) are included to demonstrate the relationship between the arthroscopic volume‐based score and histological evaluation.

A moderate positive correlation (Spearman correlation coefficient, *r*_s_ = 0.64, 95% CI 0.44–0.81, *p* < 0.001) was observed between the arthroscopic ICRS total score and the volume‐based score (Figure 3). Additionally, the volume‐based score showed a moderate positive correlation with the histological ICRS II scores, both in the overall assessment subscore (*r*_s_ = 0.62, 95% CI 0.36–0.79, *p* < 0.001) and the average of all histological subscores (*r*_s_ = 0.64, 95% CI 0.38–0.81, *p* < 0.001) (Figure 4).

## 4 Discussion

In this study, we aimed to enhance the reliability of cartilage repair assessment by introducing a standalone, volume‐based scoring method. This method dichotomously classifies repair tissue as either “good” or “poor” quality and evaluates it by quarters of the defect, ensuring a structured assessment of the entire repair site rather than a single focal point. The volume‐based method was compared with both the arthroscopic ICRS score and histological ICRS II scores to assess its reliability and alignment with established evaluation systems. Previous studies indicate that existing arthroscopic scoring systems often suffer from limited inter‐ and intrarater reliability, with increased complexity contributing to greater variability. As more tissue characteristics are incorporated, observer discrepancies become more pronounced. Additionally, the heterogeneity of repair tissue complicates evaluation, as different regions within a defect may vary in quality. By simplifying assessment to a binary classification and emphasizing volumetric evaluation rather than isolated points, our approach aims to improve reliability. Although this method is not intended as a refinement of existing scoring systems, it provides insights into how simplification can impact reproducibility and may inform future efforts to enhance the reliability of other scoring methods.

Our findings demonstrate that the volume‐based score provided moderate to good interrater reliability (ICC 0.67–0.78) and moderate to good intrarater reliability (ICC 0.58–0.84). When compared with our previous study using the ICRS score in the same model, the test score yielded higher reliability than the poor to moderate interrater reliability of the ICRS score (ICC 0.46–0.60) [5]. Similarly, previous studies assessing the reliability of the arthroscopic ICRS scoring system have reported interrater ICCs of 0.61 [14] and 0.62^2^, which align with our findings that existing methods often exhibit variability in observer agreement. These results suggest that simplifying the evaluation process may improve consistency between evaluators, supporting our hypothesis that reducing complexity leads to enhanced reliability in cartilage repair assessment. Although our ICC values indicate moderate to good reliability, further validation is needed to assess the method's robustness in broader clinical applications.

External validation of the test score was performed by comparing it to the histological ICRS II scores, often considered the gold standard for assessing cartilage repair. The volume‐based score showed moderate positive correlations with both the ICRS II overall subscore (*r*_s_ = 0.62) and the average subscore (*r*_s_ = 0.64). These correlations, though moderate, were higher than those previously observed between the arthroscopic ICRS and histological ICRS II scores (*r*_s_ = 0.50 and *r*_s_ = 0.49) [3]. This suggests that the simplified volume‐based approach does not compromise the alignment between arthroscopic and histological evaluations.

Although our results suggest that simplification can improve reliability, important limitations must be acknowledged. The small sample size and limited number of raters may impact the generalizability of the findings. Moreover, differences in evaluator experience could have influenced scoring consistency, as more experienced raters may achieve higher reliability. Future studies should assess whether additional training or standardized calibration sessions could further enhance scoring accuracy. Additionally, the simulated arthroscopy setting—although useful for standardizing conditions—may not fully replicate the complexities of in vivo arthroscopy, where factors such as joint movement, variable fluid dynamics, and restricted visibility can impact scoring reliability. These differences may limit direct translation to clinical use, emphasizing the need for future validation studies in real arthroscopic conditions. Furthermore, the study was conducted in a porcine model, which, although anatomically comparable to the human knee joint, requires clinical validation to confirm its applicability to human cartilage repair.

A key finding of this study is that although a simplified scoring method can improve reliability, it may sacrifice detailed information critical for assessing the full quality of cartilage repair. By reducing the number of evaluation points—such as tissue fill, surface quality, and border adherence—simplified approaches may overlook subtle variations that are important for distinguishing between high‐quality and poor‐quality repairs in more complex lesions. Future refinements of cartilage repair evaluation systems should aim to balance reliability and detailed tissue characteristics, potentially by integrating structured volumetric assessments or hybrid approaches that combine qualitative and quantitative measures. Modifications to existing scoring systems, such as the ICRS score, could enhance consistency while preserving clinically relevant tissue characteristics, such as integration with native cartilage and surface properties. These refinements could help to maintain scoring reproducibility without compromising the depth of information necessary for cartilage repair assessment.

To address the loss of detailed information, mapping the repair tissue in greater depth by evaluating multiple sectors or incorporating advanced imaging techniques may enhance both reliability and clinical relevance. More objective assessment tools, such as mechanical testing of cartilage stiffness [15, 16], high‐frequency ultrasound [17, 18], mechanoacoustic testing [19, 20], optical coherence tomography [21], and electromechanical testing [22, 23, 24], could improve the accuracy of cartilage repair evaluation. Although these methods have been used to assess native cartilage damage, their application for evaluating cartilage repair tissue remains limited, and they have not yet been widely validated for this purpose or implemented in clinical practice. Future work should focus on adapting and integrating these novel technologies into arthroscopic evaluations to enhance the precision and reproducibility of cartilage repair assessment.

## 5 Conclusion

This study suggests that a volume‐based scoring method focused on the evaluation of four quadrants of the repair tissue provides moderate to good inter‐ and intrarater reliability in a porcine cartilage repair model. The test score demonstrated a moderate positive correlation with histological assessment, indicating its potential utility in enhancing current arthroscopic evaluation systems. However, further refinement is needed to balance the simplicity and reliability of scoring methods with the need for a comprehensive assessment of tissue quality. Future research should focus on developing more advanced tools that provide both reliable and detailed information about cartilage repair tissue.

## Author Contributions

J.P. conceptualized and designed the study, performed experiments and surgeries, analyzed data, and drafted the manuscript. T.P. contributed to study design, performed surgeries, and critically revised the manuscript. E.S. assisted in study design, organized the animal model, performed surgeries, and reviewed the manuscript. V.M. planned the study, managed the animal model, and reviewed the manuscript. A.M. organized the animal model, ensured animal well‐being, and reviewed the manuscript. A.V. participated in study design, manuscript writing, and review. H.K. provided statistical expertise and contributed to study design. J.K. contributed to study design, manuscript drafting, and editing. I.K. led the project, oversaw planning, and contributed to manuscript writing and editing.

## Ethics Statement

The study was authorized by the Finnish Animal Experimentation Board (ESAVI/6113/04.10.07/2015) and conducted according to the ethical guidelines and regulations of the Finnish Act on the Protection of Animals Used for Scientific or Educational Purposes (497/2013) and Government Decree on the Protection of Animals Used for Scientific or Educational Purposes (564/2013).

## Data Availability

The data sets used or analyzed (or both) during the current study are available from the corresponding author upon reasonable request.
